# Molecular Insights into Leech-Derived Bioactive Compounds: Biochemical Mechanisms and Therapeutic Potential

**DOI:** 10.3390/ijms27052112

**Published:** 2026-02-24

**Authors:** Suresh Raghavi, Balakrishnan Deva darshini, Konda Mani Saravanan, Krishnan Anbarasu

**Affiliations:** 1 Department of Bioinformatics, Saveetha School of Engineering, Saveetha Institute of Medical and Technical Sciences (SIMATS), Saveetha University, Thandalam, Chennai 602105, Tamil Nadu, India; raghaviviji@gmail.com (S.R.); rabinadarshinii@gmail.com (B.D.d.); 2 Centre for Research and Innovation, AMET University (Deemed to Be University), East Coast Road, Kanathur, Chennai 603112, Tamil Nadu, India

**Keywords:** medicinal leeches, bioactive compound, leech salivary extract, wound healing, regenerative medicine, preventive healthcare

## Abstract

The bioactive compounds that are produced by leeches combine traditional and modern treatment since the saliva of the animal contains proteins and peptides with anticoagulant, anti-inflammatory, antimicrobial, antioxidant, and regenerative properties. In this review, their biochemical profile, mechanisms and clinical uses are considered with a special focus on the fact that they are utilized to combine traditional practices with the modern developments in biomedical approaches. Proteomic and transcriptomic research has recently found more than 100 bioactive molecules, such as hirudin, calin, eglins, bdellins and destabilase, which are related to the blood-feeding process and therapeutic processes. These compounds control blood clotting, control inflammatory mediators, block microbes and enhance wound healing and the development of new blood vessels. In clinical practice, leech therapy is common in the reconstruction and microsurgical practice to reduce venous congestion and enhance graft success. They are also shown to be useful in wound healing, cardiovascular health, musculoskeletal conditions and regenerative medicine, as well as emerging drug delivery systems of recombinant proteins and nanocarriers. Some of the challenges involve biological variation, infection or bleeding risks and stringent regulations on purity and standardization. Biotechnology has improved through other developments such as recombinant protein production, high-throughput omics, and nanotechnology, which will help resolve these problems, making them safe and scalable for clinical use. Altogether, leech bioactives are the prime examples of the sophisticated pharmacology of nature, which have the potential of being used as therapeutic agents in the future. The recent approach and incorporation in personalized medicine and bioengineering models reflect the leech’s capacity to address complicated illness and unmet healthcare requirements to reassert its significance in preventive medicine and recent biomedicine.

## 1. Introduction

The concept of bioactive compounds has received considerable interest in the recent past because of its versatile usage in the food industry, pharmacology, cosmetics, agrochemicals, geomedicine, nanobioscience, plant science and other related applications. This growing interest has given rise to additional research studies aimed at enhancing access to these compounds and perfecting the extraction and synthetic manufacturing process, focusing on the dynamic development of the field and its prospects [1]. Food bioactive compounds are extrinsic molecules naturally found in foods in small amounts, yet they can produce various physiological effects [2]. With global food waste reaching nearly one trillion dollars annually, there is a growing need for sustainable solutions like circular bioeconomy strategies that recover valuable bioactive compounds from agricultural by-products such as leaves, pulp, seeds, and peels [3]. Plant-derived bioactive compounds display strong antimicrobial and antioxidant activities and are increasingly being researched as eco-friendly preservatives in the food, chemical and pharmaceutical industries, emphasizing the importance of standardized and efficient extraction methods. These compounds show significant anti-inflammatory, antihypertensive, anticancer and immunomodulatory properties, aiding in the prevention and treatment of cardiovascular, metabolic and neurodegenerative diseases [4,5].

Leech therapy, also known as hirudotherapy, originated in ancient times and continues to be practiced today in both veterinary and human medicine [6]. Live leeches have been used in therapy for a long time; however, international health authorities have not formally approved or endorsed these methods. The majority of leech extract research has focused on isolating and purifying specific compounds, then assessing their biological activity against different disease models [7]. There are about 20 well-known bioactive compounds, each with its own mode of action and therapeutic significance. Among these are eglin, hirudin, Sartain, hirustatin, apyrase, calin, destabilase and hyaluronidase [8]. In hematophagous animals, salivary glands are essential because they secrete hemotoxic substances like anticoagulants at the feeding site to prevent the host’s natural hemostatic reaction. These secretions are necessary for blood-feeding leeches like *Hirudo medicinalis* to enable prolonged feeding, which lasts around 25 min and allows them to consume almost ten times their body weight in blood, followed by a protracted period of digestion that lasts for several months [9]. Previous research has shown that leech saliva extracts have anticancer properties, particularly in inhibiting the formation of malignancies such as gliomas. It is thought that bioactive components work therapeutically by altering important cellular processes linked to the onset and spread of cancer [10]. In recent years, there has been a significant advancement made in the characterization of leech-derived bioactive compounds through the application of proteomics, biochemical, analytical and molecular techniques, enabling a deeper understanding of their structural diversity and therapeutic potential [11]. Overall, leech-derived bioactive molecules are particularly noteworthy, as medicinal leech saliva contains a unique array of pharmacologically active proteins and peptides with anticoagulant, antimicrobial, and vasodilatory effects, highlighting their significant therapeutic potential and making them a key focus for molecular and biochemical research [12].

Bioactive compounds obtained from Leeches are relevant in clinical and therapeutic activities that are the intermediaries between modern biomedical research and traditional medicine [13]. Very large numbers of bioactive compounds are present in medicinal leech saliva in the form of proteins and peptides with strong anti-inflammatory, anticoagulant, vasodilatory, antimicrobial and analgesic properties [14]. The medical efficacy of leech compounds has already been demonstrated in reconstructive and microsurgery, where leeches are applied to decongest veins and improve tissue survival and blood transfer in trauma cases or transplantation [15]. Recent biochemical analyses, including Gas Chromatography–Mass Spectrometry (GC-MS), have identified various bioactive compounds in leech saliva that exhibit anticancer and antioxidant properties. However, despite their therapeutic potential, clinical application must be approached with caution due to risks like allergic reactions, secondary infections, and excessive bleeding [16]. The products derived from Leeches are a unique and valuable resource in contemporary medicine, which combines pre-modern knowledge and developments to develop new therapeutic innovations [17].

Leech-derived bioactive compounds are the focus of the current review as an exemplification of modern knowledge in therapeutic science and traditional medicine. The anti-inflammatory agents, anticoagulants, antimicrobial peptides and vasodilators are salivary compounds that show multifariously varied and beneficial clinical effects in a mechanistically diverse manner. Their established positions in microsurgery, especially in the prevention of venous congestion and improvement in tissue viability, highlight their importance in contemporary medicine. Sustainable sourcing, accurate extraction and cross-use in other industries are gaining weight in the general scenario of leech-sourced bioactives. Nevertheless, clinical translation would require scrutiny of the safety risk, including hypersensitivity reactions, bleeding and infections, and necessitate effective safety measures and standardized preparations. Taking traditional knowledge and mixing it with modern research, leech-derived bioactives can show the complexity of pharmacological nature and have significant potential in the evolution of regenerative medicine, targeted therapies, and personalized healthcare.

## 2. Review Methodology

This scoping review compiled recent research on leech-derived bioactive compounds, focusing on their biochemical profiles, therapeutic uses, and potential practical applications. The literature primarily came from databases such as Scopus, PubMed, and Web of Science, covering 2019–2025, with keywords including “medicinal leech,” “leech saliva,” “hirudin,” “antistasin,” “eglin,” “leech proteomics,” “leech-derived therapeutics,” and “hirudotherapy.” Some earlier studies published before 2019 were included for foundational context, particularly when recent data on specific compounds or mechanisms were limited. Articles were selected if they examined leech saliva composition, extraction and purification methods, molecular mechanisms, omics analyses, or therapeutic effects like anticoagulant, anti-inflammatory, antimicrobial, anticancer, or regenerative properties. Preference was given to studies with experimental validation or mechanistic insights. Exclusions included non-English articles, studies unrelated to medicinal leeches, narrative reviews without analytical synthesis, and those lacking biochemical or experimental evidence. After removing duplicates and screening for relevance, eligible studies were categorized into key themes such as bioactive compound identification, mechanisms of action, clinical and biomedical applications, and innovative biotechnological production methods.

## 3. Traditional Uses of Leeches

Leeches have historically been both objects of fascination and sources of fear in human culture, functioning as feared parasites and valued medicinal tools for millennia. The earliest recorded use of leeches is from around 1500 BCE, as shown by an Egyptian tomb painting depicting their use to combat inflammation once thought to be the underlying cause of many diseases [18]. In classical antiquity, physicians like Hippocrates and Galen used leech bloodletting to restore humoral balance by removing excess blood, which helped treat fevers, inflammation and various systemic issues [19]. This practice was deeply rooted in Greco-Roman and medieval European medical traditions, where disease was seen as a result of imbalance among the four humors of blood, phlegm, and black and yellow bile, and leeching was considered a way to restore the body’s balance [20]. At the same time, in Asian and Middle Eastern medical systems, such as Ayurveda, Unani, and Persian medicine, leech therapy remained a critical practice well after it waned in Western medicine [21,22]. The *Sushruta Samhita*, a key Ayurvedic text, offers a detailed description of Jalaukacharana (leech application), and in Sri Lankan and Indian traditional medicine, leeches have been widely used to treat inflammatory and congestive conditions [23].

In Iranian traditional medicine, scholars like Avicenna and Abdul Latif Baghdadi recognized the healing potential of leeches for up to 125 different ailments, demonstrating a sophisticated understanding of their therapeutic uses. These early physicians also recorded post-treatment care practices such as cupping the bite site to manage bleeding and avoiding cold water or air exposure to prevent swelling and infection [24]. In Unani medicine, the practice of Irsal-e-Alaq is still used to treat chronic ulcers, eczema, varicose veins and musculoskeletal pain [22]. Leeches inhabit freshwater ecosystems like ponds, lakes, and slow streams globally, except Antarctica, and include about 680 species across 91 genera. Half of continental species occur in the Holarctic, with endemism hotspots at Lake Baikal (Siberia, ~10 species) and Lake Ohrid (Balkans) [25]. Medicinal leeches (*Hirudo* spp.) show belt-shaped east–west ranges driven by ecology. *H. medicinalis* spans Britain and Norway to the southern Urals and Altai in deciduous zones; *H. verbana* spans from Switzerland and Italy to Turkey and Uzbekistan in Mediterranean/sub-boreal steppes; *H. orientalis* is found in Transcaucasia, Iran, and the Central Asia mountains; and *H. troctina* is found in Africa and Spain. In Asia, species like *Haemadipsa sylvestris* and *H. manillensis* prevail in India’s Assam damp forests [26]. Leeches are not only therapeutically valuable but also ecologically and biogeographically significant. In India alone, about 45 species spanning 22 genera have been documented, with the Himalayan areas of Arunachal Pradesh and Sikkim recognized as biodiversity hotspots [27].

Recent taxonomic research has improved our understanding of the genus *Hirudo* by identifying four main Western Palaearctic species, namely, *H. medicinalis*, *H. verbana*, *H. orientalis*, and *H. troctina*, each inhabiting separate ecological niches across Europe to Central Asia [28]. In modern medicine, Europe primarily uses *Hirudo medicinalis*, while Asia mainly relies on *Hirudinaria manillensis*. North America depends on *Macrobdella decora*, and South America utilizes *Haementeria ghilianii* and *H. officinalis* [23]. After experiencing a decline in the late 19th and early 20th centuries, partly due to advances in pharmacology and growing skepticism toward humoral theory [29], in the late 1980s, leech therapy faced a biomedical revival, particularly in microsurgery and reconstructive procedures where leeches relieve venous congestion by improving venous drainage and restoring arterial flow. The Food and Drug Administration (FDA) subsequently approved their medical use for these indications [24]. Veterinary medicine has also adopted their use, with successful cases of leech therapy resolving venous obstructions in animals [30].

Scientifically, this renaissance is largely attributed to biochemical discoveries showing that leech saliva contains a complex mix of bioactive molecules, such as anticoagulants (hirudin), platelet aggregation inhibitors (calin, saratin), anti-inflammatory enzymes (eglins, bdellins), vasodilators, antimicrobial peptides and pain-relieving substances [31]. These findings offer mechanistic validation for the therapeutic effects historically documented by ancient physicians, linking humoral-era philosophy with contemporary molecular insights [32]. Although leeches provide clinical benefits, they remain ecologically vulnerable, with medicinal populations existing at low sustainable numbers and facing threats from habitat destruction, overharvesting and temperature-dependent breeding cycles [33]. Overall, the progression from ancient Egyptian murals to FDA-approved microsurgery demonstrates how hirudotherapy exemplifies the merging of traditional and modern medicine, a testament to the ongoing link between cultural knowledge and biomedical science [34].

## 4. Biochemical Profile of Leech Salivary Secretions

### 4.1. Major Bioactive Compounds

**Figure 1:**
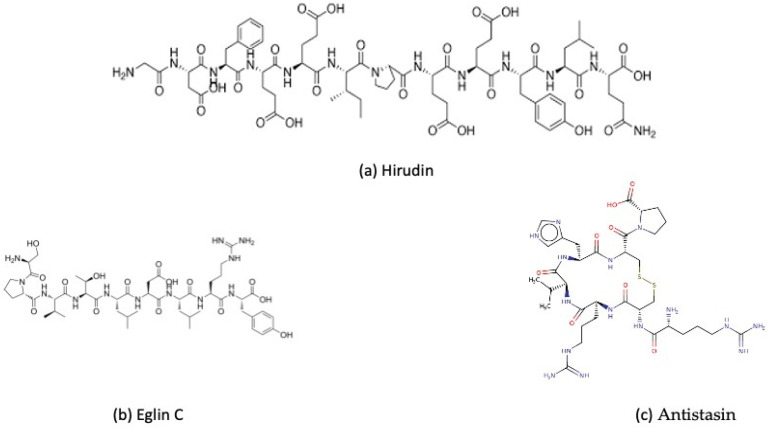
Chemical structures of key leech-derived peptides that inhibit anticoagulation and proteases. (**a**) Hirudin, a powerful thrombin inhibitor and a well-known anticoagulant; (**b**) Eglin C, a small protein that inhibits neutrophil elastase and chymotrypsin-like enzymes; and (**c**) Antistasin, which targets factor Xa and plays a role in preventing coagulation cascade progression. These compounds demonstrate the structural variety of salivary bioactive molecules from leeches responsible for anticoagulant and anti-inflammatory effects.

The methods of extraction greatly influence both the effectiveness and the long-term commercial viability of pharmaceuticals derived from leech extracts. Furthermore, comprehensive characterization of the extract components is essential for advancing their development as a therapeutic agent [35]. The genus *Hirudo* contains some of the most well-studied medicinal leeches, including species from Europe (*Hirudo medicinalis* and *H. verbana*), North America (*H. troctina*) and Asia (*H. nipponia* and *H. tianjinensis*), all of which are found in different parts of the Palaearctic and its surrounding regions [36,37]. Glossiphoniidae occur across Eurasia and into the Arctic, exhibiting cryptic diversity and broad geographical distributions. This includes Arctic endemics such as *Glossiphonia arctica* and *Hyperboreomyzon polaris*; while mussel-associated species in East and Southeast Asia, East African species display regionally distinct assemblages [38,39]. *Hirudo asiatica, Hirudo manillensis, Hirudo michaelseni, Hirudo orientaliss, Macrobdella decora* and *Hirudo granulosa* are some of the other most widely used medicinal leeches [40]. Recent research on leech saliva has revealed a diverse array of bioactive compounds and proteins, including antiplatelet factors (calin, saratin, antithrombin agents like hirudin, bufrudin) and antimicrobial compounds (theromacin, theromyzin) [41]. Around 20 bioactive molecules are well characterized with established mechanisms of action and therapeutic significance. In total, more than 100 such compounds have been identified, although many remain poorly understood concerning their chemical structures, mechanisms, and clinical applications [42]. Figure 1 illustrates representative structures of key bioactive compounds from leech saliva, such as hirudin, eglin C, and antistasin. These peptides are structurally diverse and target thrombin, factor Xa, and inflammatory proteases, contributing to anticoagulant and anti-inflammatory effects. The main bioactive compounds, their sources, extraction techniques and functional domains are summarized. Structural classification is based on reported sequence length, tertiary structure, and functional domain. Small folded inhibitors (mini-proteins) are categorized as proteins even if their molecular weight (5–15 kDa) overlaps with peptides because molecular mass is not a reliable way to distinguish leech salivary bioactive compounds (Table 1).

A study was performed to sequence the genome of *Whitmania pigra* and compare it with those of *Hirudo medicinalis* and *Helobdella robusta*. The 177 Mbp genome contained 23% repetitive sequences and 26,743 predicted genes with 46% and 38% orthologs shared with *H. medicianis* and *H. robusta*. Synteny analysis revealed 24% and 20% alignment, and a novel hirudin variant (hirudin_2) with a unique cysteine pattern was identified [51]. Another study profiled the leech salivary gland using interfrated proteomics and transcriptome analyses, indexing 434 full-length sequences, including 44 proteins and 221 transcripts linked to feeding-related bioactive pathways. Gene expression data indicated that many of these molecules are essential for feeding behavior and species-specific adaption, highlighting variability among leech species and underscoring the value of high-throughput omics approaches for discovering low-abundance therapeutic proteins [52]. Another study discovered bioactive compounds like gibberellic acid and a methoxyphenyl-chromen derivative that strongly bind to anti-apoptotic proteins Bcl-2 and Survivin. The therapeutic potential of *Hirudo vebana* saliva was evaluated in endothelial and ovarian cancer cell models using GC-MS, the Bradford protein assay, antioxidant testing, and metabolic viability testing, which showed a 63.02% viability reduction at 50% concentration, supporting its further investigation as an anticancer investigation [53].

### 4.2. Extraction Techniques

**Figure 2:**
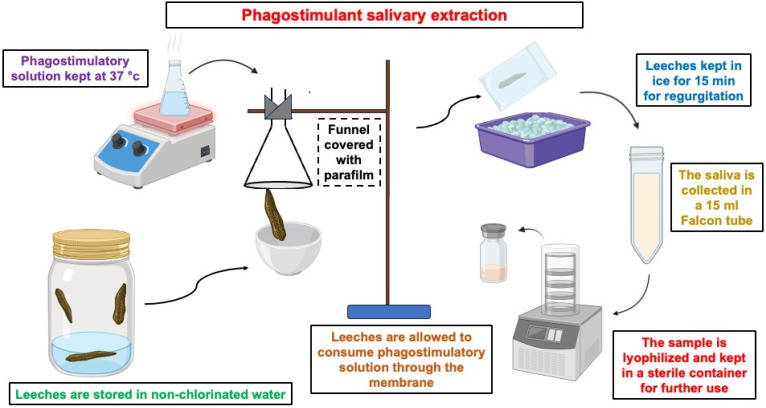
Phagostimulant-Induced Salivary Extraction Protocol from Medicinal Leeches.

Recent advances in isolating medicinal leech salivary secretions have focused on refining techniques to ensure reproducibility, improve purity, enhance productivity and advance animal welfare [13,33]. The conventional and well-developed method of preparing leeches involves starving them for around three months, which allows their digestive systems to remove any leftover blood and ensures that the extracted saliva is as pure as possible [53]. In many protocols, leech saliva is collected using an artificial feeding system in which leeches puncture a parafilm-covered membrane over a pre-warmed phagostimulatory saline solution, sometimes supplemented with arginine to enhance secretion. The regurgitated saliva is collected, and residual secretions are obtained by gentle stroking, yielding a clear, blood-free extract suitable for biochemical analysis [54]. Leeches are kept in non-chlorinated water and allowed to ingest a warm, phagostimulatory solution through a membrane. Inducing regurgitation involves cold treatment, and saliva is then collected in sterile tubes and lyophilized for storage. This approach allows for non-destructive collection of salivary bioactive compounds while keeping the leeches alive for ongoing research use. The Phagostimulant-Induced Salivary Extraction Protocol from Medicinal Leeches is detailed in Figure 2.

Another alternative method for saliva extraction is to rinse leeches with distilled water first and then gradually administer an 8% (*v*/*v*) ethanol solution. Leeches are highly sensitive to ethanol, which induces regurgitation and saliva secretion within 15 min. To remove contaminants and obtain a pure salivary sample, the collected secretion is further purified by a series of centrifugation and filtration steps [55]. Saliva from *Hirudo verbena* and *Hirudo orientalis* obtained from clinical and research sources in Baghdad was used in one study. Leeches were starved for 5-6 weeks in non-chlorinated water with regular changes, and saliva was collected using a parafilm-covered funnel containing pre-warmed 0.15 M with arginine 0.001 M to simulate feeding. After feeding, regurgitation was induced by brief cooling, and residual secretions were collected by gentle compression. The saliva was clarified by low-temperature centrifugation at 2800 rpm, yielding protein-rich extracts for further analysis [56]. Table 2 summarizes the main experimental strategies for extracting leech salivary bioactive compounds, emphasizing their benefits regarding yield, purity, reproducibility, and sustainability. This comparison offers readers practical insights into how various extraction methods impact subsequent biochemical analysis and drug development.

**Figure 3:**
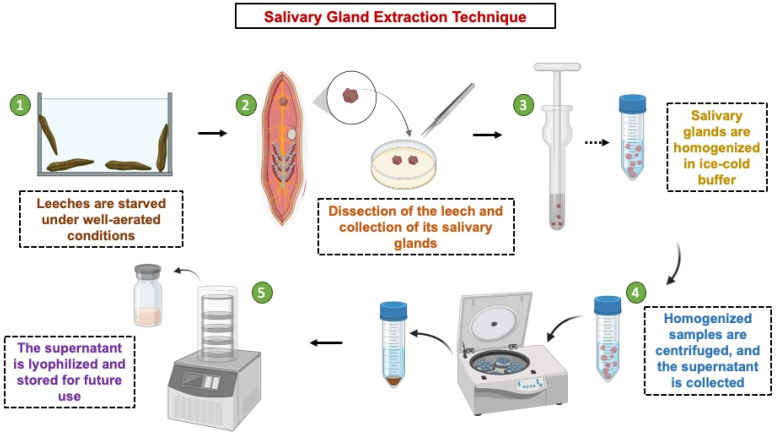
Protocol for Salivary Gland Extraction and Processing from Medicinal Leeches.

Alternative techniques for extracting leech saliva have been documented, especially where the use of an artificial feeding system is not feasible due to physiological and technical constraints. Leeches are mildly sedated using either ice or anesthetics for a brief period of time in these methods, and their saliva is manually expressed by gently milking their bodies [57]. In certain protocols, salivary glands are excised post-mortem, after which the dissected tissues are homogenized either mechanically or enzymatically to release their contents, followed by purification through sequential filtration and centrifugation. One study performed a histological examination of the glandular composition of adult leeches exposed to a relaxation buffer containing 4.8 mM NaCl_2_, 10 mM MgCl_2_, 1.2 mM KCl and 8% ethanol [58]. Leeches are starved under aerated conditions, dissected to isolate salivary glands, which are homogenized in ice-cold buffer, and then centrifuged, and the supernatant is lyophilized for further biochemical analysis. This method enables the extraction of gland-specific proteins like hirudin, eglins, bdellins and antistasin. The protocol for extracting and processing salivary glands from medicinal leeches is described in Figure 3. Adult leech salivary glands examined by scanning electron microscopy (SEM) showed a structure made up of unicellular glands arranged in clusters that resembled grapes. These glands had well-developed cell aggregates connected by delicate canalicular networks, and they had spherical, pear-shaped and ovoid morphologies of various sizes [59]. A study on *Whitmania pigra* was conducted by processing dried bodies into powder, sieving and extracting using 0.9% saline solution. The residues were then extracted twice with a greater yield. A THR-based affinity chromatography technique was combined using hirudin as a positive control in order to find thrombin (THR) inhibitors. By using this method on leech extracts, 34 peptides were found, six of which, according to a literature screening, may have antithrombin activity. The activity of these peptides was confirmed by their synthesis and testing using in vitro enzymatic tests. The discovery of SYELPDGQVITIGNER, a new antithrombin peptide with an IC50 of 255.75 μM in *W. pigra* and *H. nipponica*, was reported. Molecular docking demonstrated that its inhibitory effect was mediated by hydrogen bonds, electrostatic interactions and van der Waals forces, indicating strong anticoagulant potential [60].

**Table 2:** Approaches for leech saliva collection and purification and their advantages.

Description	Method	Saliva Collection Approach	Purification	Advantages	References
Leeches fed saline with parafilm membranes	Artificial feeding	Regurgitated saliva collected from membranes	Centrifugation and filtration	Reproducible, controlled collection	[61]
Leeches starved for 3 months to clear digestive system	Starvation method	Natural secretion harvested	Clarification by centrifugation	Produces pure, blood-free saliva	[62]
Leeches fed until full and then frozen and gently pressed	Freezing–stroking	Manual expression of saliva from leech body	Cold centrifugation	High protein content	[63]
Rinsing with distilled water followed by ethanol exposure	Ethanol-induced	Ethanol causes regurgitation of saliva	Centrifugation to remove contamination	Efficient secretion induction	[64]
Postmortem removal of glands	Salivary gland excision	Glands homogenized mechanically or enzymatically	Filtration and centrifugation	Direct extraction	[65]
Mild sedation by ice/anesthetic manual milking	Manual milking	Expression of saliva by stroking and milking	Sequential filtration and centrifugation	Alternative when artificial feeding not feasible	[66]
Dried leech bodies powdered and extracted with saline	Powder extraction	Extraction of bioactives from powder	Affinity chromatography	Enables isolation of specific peptides	[67]
Combine gland proteome with transcriptome analysis	Proteomic transcriptomic integration	Identification of low-abundance bioactives	Bioinformatics and LC-MS/MS	Comprehensive bioactive profile	[68]

### 4.3. Structure and Function

The therapeutic uses of leech saliva are supported by a wide range of bioactive compounds with distinct structural and functional properties that reflect evolutionary adaptations for effective hematophagy [69]. Enzymes, proteins and peptides are among the bioactive compounds found in leech secretions. Key compounds such as growth factors, anticoagulants and antimicrobial peptides contribute to the therapeutic effects of leech saliva by enhancing skin hydration, promoting wound healing and reducing inflammation. Effective application of these bioactive compounds requires targeted delivery to the affected area [70]. Hirudin, with a molecular weight of 6.97 kDa, is a 65-amino acid protein with the sequence VVYTDCTESGQNLCLCEGSNVCGQGNKCILGSDGEKNQCVTGEGTPKPQSHNGDGFEEIPEEYLQ. Hirudin from *Hirudo medicinalis* is a bivalent thrombin inhibitor with strong anticoagulant properties that target both the active site and exosite of thrombin at the same time [71]. Hirustasin is a 55-amino acid polypeptide with a molecular weight of 6.08 kDA, with the sequence TQGNTCGGETCSAAQVCLKGKCVCNEVHCRIRCKYGLKKDENGCEYPCSCAKASQ.

Its functions include inhibiting serine protease and binding to kallikreins, which are a subset of serine proteases found in tissues and plasma that cleave kininogens to produce kinins [72]. The amino acid histidine is decarboxylated to form histamine (C_5_H_9_N_3_), a biogenic amine with an imidazole ring. During feeding, leech salivary glands release histamine, which causes the smooth muscles and blood vessels at the bite site to partially relax [73]. Functionally, these compounds facilitate effective blood feeding and underpin the therapeutic potential of medicinal leech therapy by mediating a variety of physiological consequences. Hirudin prevents blood clots by directly blocking thrombin, thereby exerting anticoagulant action [74]. Anticoagulants such as guamerin and antistasin preserve blood fluidity at the leech feeding site by acting on different stages of platelet aggregation and coagulation pathways [75]. Pharmacological targets, mechanisms, and therapeutic uses of key leech salivary compounds are listed in Table 3.

The i-type lysozyme destabilase from *Hirudo medicinalis* has isopeptidase and muramidase activity, both of which are inhibited by physiological sodium levels. One study identified two crystal structures, including a sodium-bound version at 1.1 Å resolution, that showed coordination at Glu34/Asp46 and most likely explained the inhibition of muramidase. His112, not Lys58, is the universal base for isopeptidase activity, according to sequence comparison and pKa estimates based on molecular dynamics. These findings shed light on the catalytic mechanisms of destabilase and offer a structural foundation for further research into its isopeptidase function and possible anticoagulant medication development [76]. Leech saliva contains over 100 biologically active proteins, including an antimicrobial that was investigated for its activity against *Streptococcus mutans* and *S. sobrinus*, key contributors to tooth decay. Saliva samples were collected from three leech groups at one, two, and three months post-feeding, and antimicrobial properties were assessed alongside bioinformatics and molecular docking analyses of destabilase. Protein concentration increased with starvation duration, reaching 776 μg/mL after three months. Saliva from three-month starved leeches exhibited the strongest antimicrobial effects, with 35.3% inhibition of *S. mutans* and 42.6% of *S. sobrinus* at 2 mg/mL, and the highest anti-biofilm activity (31.6% and 44.2%, respectively). These findings highlight destabilase as a promising agent for preventing and treating dental caries [62].

**Table 3:** Pharmacological targets, mechanisms and therapeutic applications of major leech salivary compounds.

Compound	Target Pathways/Molecules	Pharmacological Properties	Mechanism of Action	Therapeutic Application	Safety/Limitations	References
Hirudin	Thrombin active/exosite sites	Potent anticoagulant	Direct thrombin inhibition	Stroke prevention, reconstructive surgery, cardiovascular disease	Risk of bleeding, hypersensitivity	[77]
Eglin C	Elastase, chymase enzymes	Anti-inflammatory	Elastase, chymase inhibition	Osteoarthritis, tissue regeneration	Low toxicity	[78]
Bdellin	Trypsin, plasmin	Anti-inflammatory, protease inhibition	Protease inhibition	Chronic inflammation, rheumatoid arthritis	Mild allergies	[42]
Destabilase	Bacterial cell wall, fibrin	Anticoagulant, antimicrobial	Peptidoglycan degradation and anticoagulant	Infection control, wound healing, dental caries	Limited systemic data	[79]
Calin	Platelet adhesion molecules	Antiplatelet, antithrombotic	Blocks platelet collagen interaction	Venous congestion, flap salvage in microsurgery	May increase hemorrhage risk	[80]
Saratin	Platelet collagen binding	Antithrombotic	Inhibits platelet adhesion	Ischemic tissue rescues, reconstructive surgery	Occasional local reactions	[81]
Hirustasin	Kallikrein, factor Xa	Anticoagulant, anti-inflammatory	Serine protease inhibition	Inflammatory disorders, blood pressure regulation	Allergy potential	[82]
Antistasin	Factor Xa	Anticoagulant	Inhibits coagulation factor Xa	Treatment of deep vein thrombosis, myocardial infarction	Bleeding risk	[83]
Theromacin	Bacterial and fungal membranes	Broad spectrum antimicrobial	Membrane disruption of microbes	Management of bacterial infections	Potential cytotoxicity at high doses	[84]
Hyaluronidase	Facilitates tissue permeability, drug diffusion	Extracellular matrix degradation	Hyaluronic acid	Enhances drug delivery, wound healing	Tissue damage with prolonged exposure	[85]

## 5. Mechanism of Leech-Derived Bioactives

### 5.1. Anticoagulant

Anticoagulants lower the risk of thromboembolic disorders, such as stroke, deep thrombosis and myocardial infarction, by either preventing blood clot formation or delaying the clotting process. Hirudin, heparin, streptokinase, antithrombin and urokinase are examples of naturally occurring compounds. Several naturally occurring anticoagulant proteins are used to treat thrombotic conditions [86]. A variety of medicinal plants have been identified as potential sources of anticoagulant agents, including *Allium sativum* (garlic), *Persea americana* (avocado), *Erigeron canadensis*, *Piper betle* and *Tridax procumbens*, all of which have demonstrated significant anticoagulant properties [87]. Rosaceae and *Fabaceae Lamiaceae* are other plant families with notable anticoagulant properties [88]. The most widely used anticoagulant medicine is heparin, which is derived from animal tissue such as the intestine of pigs, sheep and cows [89,90]. Proteins and peptides extracted from a variety of sources, including bee venom and snake venom, are examples of animal-derived anticoagulants. Anticoagulants derived from venom primarily work by blocking key enzymes in the coagulation cascade [91]. Blood-feeding leeches possess potent anticoagulants that allow them to feed for extended periods. Whether they express these proteins and whether they are appropriate for other uses are unknown [92].

Cardiovascular disease continues to drive the search for safer natural anticoagulants. A study isolated a thermostable anticoagulant protein, WP-77 (20.8 kDa), from the non-hematophagous leech *Whitmania pigra* through chromatographic purification. WP-77 remained stable across pH 2–8 and temperatures of 20–100°C, retained activity after pepsin treatment, and promoted endothelial repair. It significantly prolonged APTT and TT without affecting PT or fibrinogen levels, and it reduced thrombus formation in vivo, indicating its potential as a novel anticoagulant candidate [93]. Leeches have developed a variety of anticoagulant techniques to overcome host hemostasis. The leech *Limnobdella Mexicana*, which consumes the mucosal membranes of mammals, sheds light on the connection between anticoagulant diversity and host range. The purpose of another study was to create the first transcriptome of a proboscis leech and describe its range of anticoagulants. Using BLAST analysis, amino acid conversion, the Pfam domain and phylogeny-based inference, 15 potential anticoagulant proteins were identified. These discoveries add to our understanding of the evolution of anticoagulants in mucosal-feeding leeches and offer a foundation for cross-taxonomic comparisons [94]. Another study investigated conformationally restricted synthetic cyclic analogs of hirudin-derived antithrombotic peptides designed through disulfide bond cyclization to inhibit fibrinogen cleavage by targeting a non-enzymatic thrombin-binding site. The cyclic peptides were evaluated using in vitro clotting assays, and analogs based on an α-helical conformational model retained significant antithrombin activity. In particular, [D-Cys58, Cys61]-hirudin54-65 and [D-Cys60, Cys63]-hirudin54-65 showed IC_50_ values of 26 µM and 30 µM, respectively, compared with 3.7 µM for the linear hirudin54-65 peptide, confirming that structural cyclization can preserve anticoagulant activity while enhancing conformational stability [95].

### 5.2. Anti-Inflammatory

Anti-inflammatory agents are compounds that, by modifying or inhibiting specific inflammatory pathways, reduce inflammation, a defense response characterized by heat, redness, swelling and discomfort [96]. Restoring tissue homeostasis is facilitated by their modes of action, which include downregulating pro-inflammatory cytokines like IL-6 and TNF-α, inhibiting important enzymes like COX-1 and COX-2, and suppressing oxidative stress and immune cell recruitment [97]. Anti-inflammatory agents originate from both plant and animal sources. Plant compounds that inhibit mediator synthesis, modulate gene expression, and scavenge reactive oxygen species include curcumin, polyphenols, resveratrol, gingerols, and flavonoids from herbs, teas, and fruits [98,99]. Animal-derived agents such as fish oils and omega-3 fatty acids also reduce inflammation by shifting eicosanoid synthesis towards less pro-inflammatory pathways [100]. In contrast, leech saliva is a complex mixture containing nearly 100 bioactive molecules, such as hyaluronidase, hirudin, eglins, destabilase, and beldins, which collectively exert anticoagulant, anti-inflammatory, analgesic and vasodilatory effects through complementary mechanisms [99]. This multi-component activity may provide advantages over single-compound plant or animal bioactives by simultaneously targeting proteases, coagulation pathways, microcirculation, and cytokines. For example, eglin C inhibits inflammatory proteases, like chymase, elastase, cathepsin, and subtilisin, while bedellins inhibit proteases, including acrocin, trypsin, and plasmin [14,101]. Leech salivary components also reduce interleukin-6 and tumor necrosis factor levels in affected tissues, supporting cytoprotection and the resolution of inflammation. Overall, these diverse biotherapeutics offer a multifaceted approach to managing inflammation [102].

The bioactive compounds in leech saliva that possess anti-inflammatory, antimicrobial and analgesic properties have long been utilized in both traditional and modern medicine. One study aimed to identify the molecular components of lyophilized leech saliva, evaluate their effect on inflammation, and explore their potential as a biotechnological anti-inflammatory drug. The extract was tested on in vitro-activated mammalian macrophages after its peptide and protein contents were identified using LC-MS/MS and proteomics studies. The therapy drastically reduced the production of pro-inflammatory cytokines, suggesting it could be used as a treatment for autoimmune and inflammatory diseases [103]. Another study examined hirudin (Hiru) for its protective effects on rat vascular smooth muscle cells (VSMCs) in a chronic renal failure (CRF) rat model, aiming to prevent vascular injury and inflammation. Blood urea nitrogen and serum creatinine levels were used to assess renal function in CRF rats given different doses of Hiru. H&E staining was employed to evaluate vascular damage, while immunohistochemistry examined iNOS and Arg-1 expression. Western blotting assessed macrophage polarization, apoptotic markers and proliferation, whereas ELISA analyzed inflammatory cytokines. VSMC viability was evaluated using the CCK-8 test. Hirudo therapy showed potential as a therapeutic agent against CRF-associated vascular injury by reducing inflammatory responses [104].

### 5.3. Antimicrobial and Antioxidant Activity

Antimicrobial activity refers to a substance’s capacity to inhibit growth or eradicate microorganisms, such as bacteria and fungi [103]. Antioxidants are compounds that scavenge free radicals and reduce oxidative stress, thereby helping maintain health, prevent disease, and stabilize food [104]. Bioactive compounds found in herbs, fruits, spices and vegetables, including flavonoids, polyphenols, tannins, saponins and essential oils, are richly stored in plants. These compounds have antimicrobial properties that promote health protection when consumed by humans [105]. The Musaceae family, which includes banana, is one of the most extensively grown fruit crops in the world and one of the first known medicinal plants. Its bioactive compounds, especially antimicrobial agents, have been the main focus of recent research. The antimicrobial properties of bananas include ferulic acid, lupeol, 3-carene, gentisic acid and dopamine [106,107]. In a similar way, animal-derived compounds that strengthen innate immune responses and protect tissues, such as lysozyme from egg whites, defensins, antioxidant enzymes and peptides, are crucial to these processes [108,109]. A variety of bioactive peptides and enzymes, including hirudin, destabilase and other antimicrobial peptides, are abundant in leech saliva and have potent antimicrobial activity by disrupting bacterial and fungal membranes [110]. In addition to its antimicrobial potential, leech saliva also exhibits antioxidant properties mediated by enzymes and peptides that scavenge free radicals and activate endogenous antioxidant systems, thereby supporting anti-inflammatory and wound healing mechanisms [111,112].

A study was conducted to assess the antimicrobial activity of leech salivary extract (LSE) and its silver nanoparticle formulation (LSE-Ag) against *Pseudomonas aeruginosa*, *Escherichia coli*, *Staphylococcus aureus* and *Klebsiella* spp. using agar well diffusion and microbroth dilution methods. LSE-Ag was biologically synthesized and characterized (UV absorbance at 456nm, particle size 98.04 nm), and the findings revealed that while crude LSE exhibited no activity, LSE-AG significantly inhibited *P. aeruginosa* (8.3 ± 0.88 mm and 12.3 ± 0.88 mm) and *Klebsiella* spp. (12.0 ± 0.57 mm and 12.3 ± 0.33 mm) at 100 µL, with MIC at 100 µL and MBC at 200 µL, whereas *S. aureus* and *E. coli* remained resistant, indicating the potential of LSE-Ag in managing infections caused by *P. aeruginosa* and *Klebsiella* spp. [113]. Another study on Indian cattle leech *Poecilobdella granulosa* examined the biochemical composition and antioxidant potential of crude extracts from different organs. Protein and carbohydrate contents were quantified, protein profiles were analyzed by Native and SDS-PAGE and free radical scavenging activity was assessed. The whole-body extract showed the highest protein and carbohydrate levels, with bands ranging from 22 to 110kDa, whereas only the salivary extract exhibited significant antioxidant activity in DPPH and superoxide assays, indicating *P. granulosa* saliva may serve as a selective natural antioxidant with potential health applications [114].

### 5.4. Angiogenesis and Tissue Regeneration

**Figure 4:**
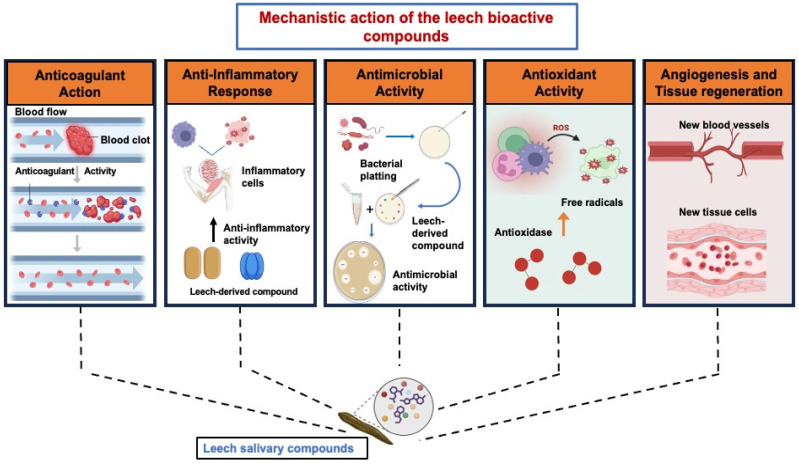
Proposed Mechanism of Action of Bioactive Molecules from Medicinal Leeches.

Angiogenesis is a basic biological process that is necessary for wound healing, tissue regeneration, organ repair and integration of transplanted or manufactured tissues. It is described as the creation of new blood vessels from pre-existing vasculature [115]. It is a strictly controlled process that involves the development of new blood vessels, which is essential for both healthy physiological processes and pathological circumstances, including the development and metastasis of tumors [116]. During tissue repair, the initially formed vessels undergo remodeling and maturation, giving rise to a stable vascular network that sustains long-term tissue regeneration and structural remodeling [117]. Over the past three decades, natural compounds or their structural variants have been the source of around 80% of approved angiogenesis-related medications [118]. A carotenoid found in brown seaweeds, fucoxanthin, controls angiogenesis by preventing endothelial progenitor cells from differentiating and by inhibiting the development of blood vessel-like structures. It also exhibits a dose-dependent reduction in microvessel sprouting together with its derivative fucoxanthinol, indicating its potential as a natural angiogenesis inhibitor [119]. Leech saliva extract (LSE) exhibits anti-angiogenic properties by promoting cleaved caspase-3 production and inhibiting endothelial cell adhesion and angiogenesis. Furthermore, LSE inhibits the VEGFA signaling pathway’s integration into liposomal delivery vehicles, enhancing its inhibitory effect on angiogenesis [53]. Leech salivary molecules have various pharmacological effects, such as anticoagulant activity through thrombin and factor Xa inhibition, anti-inflammatory effects by modulating cytokines and proteases, antimicrobial actions, antioxidant scavenging of reactive oxygen species and promotion of tissue regeneration via angiogenesis. These mechanisms support their therapeutic potential in wound healing, cardiovascular disorders, and reconstructive surgery (Figure 4).

A study was conducted to investigate whether leech extract promotes angiogenesis through endothelial cell-derived effects (EC-EX). In ischemic stroke, the objective is to explore its role in collateral circulation establishment and underlying mechanisms using an in vitro EC-pericyte co-culture system and an in vivo mouse model of middle cerebral artery occlusion. It was found that LSE enhanced pericyte proliferation and migration, reduced infarct area, improved repair and activated the HIFα-VEGF-DLL4-Notch1 signaling pathway, thereby promoting cerebral angiogenesis and stabilizing neovascular maturation, suggesting that its effects may be mediated by modulation of exosomal miRNA and angiogenesis-related signaling pathways [120]. Another study assessed the anticancer effects of lyophilized leech saliva extract (LLSE) on MDA-MB-231 breast cancer cells and HUVEC endothelial cells by testing cell viability, migration, apoptosis, and gene expression. LLSE showed selective toxicity to cancer cells, with an IC_50_ of about 470 µg/mL, while promoting growth in healthy endothelial cells, with measurable EC_50_ values, indicating a dose-dependent, differential effect. The treatment decreased cancer cell migration and caused necrosis while influencing key angiogenic factors like FGF, VEGF, and EGF. This suggests that LLSE could act as a dual-function therapeutic, simultaneously inhibiting tumor growth and supporting normal endothelial repair mechanisms [63].

## 6. Clinical Applications

### 6.1. Wound Healing and Diabetes

The wound healing process is a multi-phase, intricate event that includes remodeling hemostasis, proliferation and inflammation. It is controlled by a variety of mechanisms that react to and adjust to dynamic changes in broken tissue [121]. Diabetes causes slow healing and infection-prone sores, such as diabetic foot ulcers, due to chronic hyperglycemia, neuropathy, vascular damage, ongoing inflammation that impairs circulation, immunological function, and angiogenesis [122]. Natural bioactive compounds from plants and animals have pro-angiogenic, antimicrobial, antioxidant and anti-inflammatory properties that may be used therapeutically to treat diabetic wound healing [123]. Phytochemicals like alkaloids, flavonoids, phenolic compounds and saponins are mainly responsible for the wound healing properties of many herbal medications, according to research. Although clinical safety assessments are still necessary for wider use, incorporating natural compounds into nanotechnology-based delivery systems may enhance their therapeutic efficacy [123]. Bioactive phytochemicals increase keratinocyte and fibroblast activity, improve collagen deposition, stimulate vascularization to reduce oxidative stress, aid in wound healing and control inflammatory pathways [124]. The most widely used leech species for therapeutic purposes is *Hirudo medicinalis*, while other species have also been studied worldwide [125]. Leech therapy has been used to improve wound healing, especially in chronic non-healing wounds and post-traumatic wounds. Studies in rats and mice have shown that increasing local blood flow with hirudotherapy improves flap survival and reduces necrosis. When researchers evaluated ischemic preconditioning, leech treatment dramatically decreased necrosis and raised flap survival in rats, which averaged 88% [126,127].

A study was conducted to evaluate the effect of leech therapy on angiogenesis in chronic non-healing ulcers with a focus on vascular endothelial growth factor (VEGF) expression. Thirty patients were divided into two groups in the pilot randomized clinical trial. One group was given normal saline and Jatyadi Ghrita dressing to clean their wounds, and the other group also received leech therapy on days 0, 7, 14, 21 and 28 in addition to oral Ayurvedic medications [128]. Another study used lyophilized *Hirudo orientalis* to create a 5% cream that contained bioactive proteins that had anticoagulant and wound healing qualities. The formulation’s anticoagulant effect was validated by its significant increase in partial thromboplastin time, the lack of microbiological contamination and its excellent skin absorption. Similar to phenytoin, the leech cream aided re-epithelialization and angiogenesis in in vivo studies, indicating that it could be a safe and effective alternative to conventional wound healing treatments [129]. Using a rat excision model, a study investigated the effect of medical leech therapy (MLT) on wound healing. Three groups were formed from thirty rats with full-thickness dorsal wounds. Notable differences in wound contraction were seen in biopsies taken on days 6 and 16, with the MLT group showing faster healing and less inflammation than the controls. Compared to traditional treatment, medical leech therapy demonstrated better overall healing in excisional wound repairs [130].

### 6.2. Reconstructive Surgery and Microsurgery

Reconstructive and microsurgical techniques are essential in plastic surgery because they restore both function and appearance to body parts damaged by trauma, congenital anomalies, or tumor removal [131]. Reconstructive surgery encompasses various procedures aimed at repairing anatomical defects and restoring function [132]. The mechanisms involve anticoagulation, vasodilation, and bloodletting, all facilitated by bioactive compounds in leech saliva. In plastic surgery, hirudotherapy involves pre-treatment preparations, careful application of leeches, monitoring during the procedure, and assessing possible contraindications [133]. Microsurgery is a modern subspecialty that uses operating microscopes, ultra-fine sutures and specialized micro-instruments to repair or reconstruct tiny structures, like blood vessels and nerves, which can be as small as one millimeter [134]. Animal sources are vital for the development and application of reconstructive and microsurgical techniques [135]. Live models such as laboratory rats and rabbits are crucial for microsurgical training and research due to their anatomical similarities. They enable surgeons to develop precise motor skills and refine new surgical techniques before their clinical applications [136]. Plant-derived materials are a newer development and primarily function as alternatives for producing bioengineered scaffolds, synthetic vascular tubes, or nerve guides [137]. These plant-derived biopolymers are appreciated for their biocompatibility and are under investigation for their potential to decrease dependence on animal tissues, although their use in live surgical procedures remains largely experimental [138]. In 1980, plastic surgeons significantly advanced medicinal leech therapy by using leeches to alleviate venous congestion, particularly in transplant surgeries. These leeches can help manage tissues with venous insufficiency by providing temporary venous outflow until new blood vessels form in the graft. In July 2004, the FDA approved leeches as a medical device for use in plastic and reconstructive surgeries [139].

A comprehensive search of PubMed and Embase up to November 2023 identified 18 studies, 14 case reports and four case series covering a total of 28 patients. The JBI Critical Appraisal Checklist for Case Series was used to assess bias. The majority of patients (75%) underwent reconstructive breast surgery, with leech therapy typically involving a median of two leeches, three sessions daily, for three days. Overall, leeching successfully salvaged 75% of compromised tissue transfers, although complications were common in 81.14% of cases, primarily infections and anemia [140]. A retrospective study examined 148 patients treated with medicinal leech therapy from 2005 to 2010 to manage venous congestion after reconstructive surgeries involving local, pedicled, or microvascular flaps. All patients exhibited signs of flap congestion despite initial treatments, including suture removal, pressure relief, and hematoma evacuation. Leech therapy was used as a salvage tactic. The results showed excellent and consistent healing, especially in local and microsurgical flaps, emphasizing leech therapy’s role as a dependable adjunct in maxillofacial and reconstructive plastic surgery to combat postoperative hemodynamic issues and venous insufficiency [141].

### 6.3. Cardiovascular and Hematologic Disorders

Cardiovascular and hematologic disorders encompass a wide range of diseases that impact the heart, blood vessels and cellular or plasma components of blood [142,143]. Cardiovascular disorders encompass hypertension, coronary artery disease, myocardial infarction, arrhythmias, congenital heart defects and other heart conditions [144,145]. Hematologic disorders refer to diseases that impact components such as red and white blood cells, platelets, blood clotting factors or hemoglobin. Examples include anemia, hemophilia, sickle cell disease, thalassemia, leukemia and conditions involving abnormal clotting or bleeding [146]. Natural compounds derived from plants and animals have become increasingly prominent in the prevention and supportive treatment of cardiovascular disorders owing to their bioactive phytochemicals and secondary metabolites [147,148]. Herbal medicines like Digitalis (from *Digitalis purpurea*) continue to be important in standard heart failure treatment because of their positive inotropic effects. Additionally, reserpine (from *Rauwolfia serpentina*) also plays a role [149,150]. Animal-derived compounds offer significant anticoagulant or antithrombotic effects, including omega-3 polyunsaturated fatty acids from fish oil that influence platelet activity and inflammation [151]. Hematophagous animals secrete saliva that contains a mixture of bioactive molecules, mainly proteins and peptides. There have been notable advances in creating drugs from these animals to treat cardiovascular diseases. Examples include antihypertensive medications, such as captopril and enalapril, antiplatelet agents, such as tirofiban and eptifibatide, and anticoagulants, such as lepirudin and bivalirudin [152]. Leech proteins work together to enhance microcirculation, lower blood viscosity, prevent both local and systemic clotting and alleviate tissue hypoxia [153]. Hirudin, eglins, calin, bdellins, factor Xa and destabilase inhibitors each target different stages of the coagulation cascade or modulate inflammatory and vascular pathways [154].

Research has explored bioactive compounds from leeches for their potential to combat atherosclerosis and its mechanisms. Network pharmacology identified 34 active compounds and 1172 genes linked to atherosclerosis, with 89 shared targets involved in the PI3K–Akt, Rap1, PPAR, and AGE–RAGE signaling pathways. Molecular docking revealed strong binding of key compounds. In vivo mouse studies showed that leech treatment reduced plaque size and lowered myocardial SRC levels, indicating multi-target effects that modulate inflammation, oxidative stress, and lipid metabolism [155]. Additionally, the medicinal leech *Whitmania pigra* demonstrated protective effects against blood hyper-viscosity in a rat model. Treatment significantly decreased blood viscosity, improved aortic tissue structure, and had effects comparable to aspirin at moderate doses. Metabolomic analysis confirmed normalization of pathways, such as cysteine–methionine metabolism, the TCA cycle, and arachidonic acid metabolism, suggesting *W. pigra* may help manage hyper-viscosity-related cardiovascular issues [156].

### 6.4. Musculoskeletal and Inflammatory Diseases

Musculoskeletal and inflammatory diseases cover a wide range of conditions that affect bones, joints, muscles, ligaments, tendons, and surrounding tissues, often causing pain, limited movement, and a decreased quality of life [157]. These disorders can significantly impair mobility, function, and overall well-being. They affect over 1.3 billion people worldwide and are a major cause of disability and economic burden [158]. Musculoskeletal disorders include osteoarthritis, rheumatoid arthritis, gout, bursitis, tendonitis, and fibromyalgia, among others [159]. Medicinal plants contain a diverse array of pharmacologically active compounds with anti-inflammatory and antioxidant properties, making them promising options to support traditional treatments [160]. Phytochemicals found in ginger, turmeric, willow bark, borage oil and Stephania tetrandra help lower inflammation by inhibiting prostaglandins, leukotrienes, interleukins and TNF-α [161]. Animal-derived products, such as milk, provide immunomodulatory proteins, antibacterial peptides and oligosaccharides that support immune health and help reduce inflammation [162]. Meat and eggs supply essential amino acids, as well as coenzyme Q10, glutathione and omega-3 fatty acids, which play roles in muscle health, antioxidant defenses, and systemic inflammation [163]. Notably, leech saliva is a significant compound among animal extracts, with leech therapy (hirudotherapy) increasingly recognized for its effectiveness in treating chronic musculoskeletal pain, osteoarthritis, myofascial pain, and inflammatory joint conditions [164,165].

Leeches are used to treat knee osteoarthritis. A study showed that after applying leeches, all patients rapidly experienced pain relief. Their pain scores decreased markedly from an average of 4.7 on the Visual Analog Scale (VAS) to 1.3, and this improvement lasted for four weeks post-treatment [166]. Leech therapy (Jaloukavacharan) was studied for its anti-inflammatory and pain-relieving effects in rheumatoid arthritis patients, associated with Aamvata in Ayurveda. The study involved 10 sessions of leech application on alternate days, with evaluations at baseline (day 0), after three weeks (day 21) and follow-ups on days 30 and 45. Subjective symptoms like pain, swelling, tenderness and joint dysfunction were monitored, along with objective markers such as C-reactive protein (CRP) and erythrocyte sedimentation rate (ESR). The results showed significant decreases in CRP (23.54%, p = 0.0001) and ESR (10.30%), along with notable improvements in pain (57.62%), tenderness (72%), and walking ability (56.67%) and reductions in swelling, redness and local temperature. These findings suggest that leech therapy could be a valuable adjunct for alleviating symptoms and reducing inflammation in rheumatoid arthritis [167]. The medicinal leech serves as a useful model for studying the role of TLR4 and its associated products, like tumor necrosis factor (TNF-α), following activation of the peripheral immune system with either endogenous recombinant allograft inflammatory factor-1 (rHmAIF-1) or external stimuli, such as lipopolysaccharides (LPSs). Our results indicate that activated macrophages (HmAIF-1+) and granulocytes (CD11b+) produce both TLR4 and its co-receptor CD14. Furthermore, tests using a cyanobacteria-specific TLR4 antagonist, CyP, confirmed that only the TLR4 pathway was blocked, while the immune response to lipoteichoic acid (LTA) remained unaffected. These findings align with vertebrate studies, confirming that TLR4 acts as an LPS receptor, whereas the detection of LTA likely involves other pathways [168].

### 6.5. Drug Delivery and Regenerative Medicine

**Figure 5:**
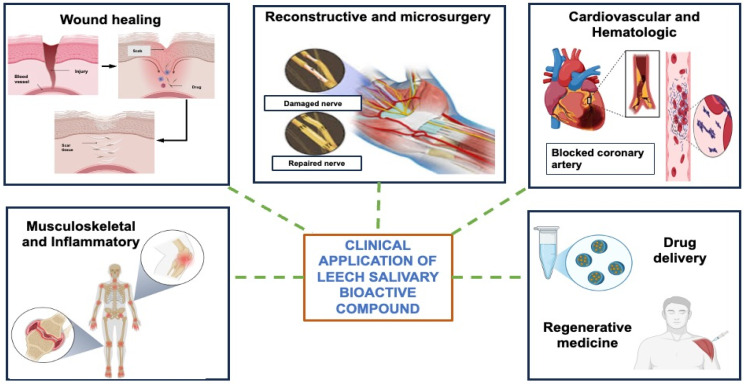
Biomedical Applications of Bioactive Compounds from Medicinal Leeches.

Modern medicine has undergone a revolution in the area of drug delivery and regenerative medicine, seeking to substitute, repair and regenerate damaged tissues or organs [169]. Regenerative medicine involves the use of scaffolds, stem cells and signaling molecules to boost the natural ability of the body to restore and repair tissues [170]. Scaffolds and substrates play an essential role in both regions to control drug release and tissue regeneration, as well as cell behavior [171]. Scaffolds used in drug delivery are valuable as they optimize the therapeutic efficacy of drugs and bioactive compounds, and they are safe and effective. They allow for directing delivery to particular tissues or organs and allow for controlling drug dosage and delivery time, which enhances the results of treatment and decreases side effects [172]. Plant-derived and animal-derived natural substances are important in regenerative medicine and drug delivery due to their abundance of bioactive molecules with potent biological activity and great biocompatibility [173]. Plant-based natural products, like polyphenols, flavonoids, alkaloids and terpenoids, possess significant antioxidant, anti-inflammatory, and immunomodulatory properties. They are appropriate for use in tissue-engineering scaffolds or as a secondary substance in stem cell therapies, in both cases, for in vivo tissue repair, cell growth and differentiation. Animal-derived proteins, peptides, and extracellular matrix materials of a range of species are successfully utilized in biomedical engineering and wound healing [174]. Leech-based extracts or single constituents have now been incorporated into scaffolds, wound coverings or injectable therapeutics to utilize these regenerative and protective effects. This underscores the huge importance of leeches in modern regenerative medicine [175]. Leech-derived molecules are utilized in reconstructive microsurgery to alleviate venous congestion, serve as anticoagulants in cardiovascular medicine, and assist in wound healing and inflammatory conditions; they are currently being investigated in regenerative medicine and drug delivery systems. The applications of bioactive compounds from medicinal leeches in biomedicine are shown in Figure 5.

To investigate the analgesic effect of leech saliva, peptides from the salivary glands of *Haemadipsa sylvestris* were studied. The research made it possible to identify a novel peptide family named HSTXs that is characterized by its distinct structures. HSTX-I was one of the peptides that was able to block NaV1.8 and NaV1.9 channels, which are important in neuronal functions and the transmission of pain. These findings affirm that leech saliva has compounds with the ability to target sodium channels and relieve pain, implying that HSTXs may possess therapeutic potential [176]. In order to overcome the shortcomings of the conventional therapies for atherosclerosis, scientists produced bowl-shaped chitosan/polycaprolactone particles through electrostatic spraying. They attained a favorable morphology of the particles by optimizing the precursor concentration and the ratio of polymer. Leech hirudin was loaded into these particles, which made it possible to release the anticoagulant slowly and under controlled degradation. The biocompatibility and hemocompatibility were high, the viability of mouse glial cells was over 90 percent, and rabbit blood hemolysis was less than 2 percent. Circulation time was improved, surface area was increased, the distribution of drugs was improved, and immune rejection was reduced when using the bowl shape. On the whole, hirudin-loaded bowl-shaped particles are an interesting platform for long-term delivery of anticoagulants, and a new blood-contacting substance that has high potential to cure vascular diseases can be developed [177].

## 7. Challenges and Future Directions

The past few years have seen an increased focus on regulatory restrictions of leech-derived bioactive compounds due to their promising therapeutic properties, but also due to the realization that natural products are not always easily solubilized in a convenient format [178]. Isolated bioactive compounds of leech saliva, particularly recombinant or purified proteins such as hirudin, are regulated instead like a biologic or drug, however. Such regulations involve extensive preclinical toxicology tests, pharmacokinetic tests, and multi-phase clinical tests in order to demonstrate their effectiveness and safety. Subsequently, this significantly raises the cost of development and timelines [175]. The issue of biological variability is a major problem; natural products that have been isolated from leech species, which are produced under various environmental factors and extracted by different methods, are quite heterogeneous between batches. Regulatory authorities insist on the uniformity of purity, potency and sterility and thus necessitate the use of superior levels of standardization processes and extensive quality assurance mechanisms [13]. Risks to safety, such as allergies, infections, and hemorrhage, need intense risk management and monitoring. Meanwhile, sustainability and animal welfare considerations regarding ethics and environmental concerns demand regulatory frameworks that support therapeutic advancements as conservation initiatives [179].

Biotechnological and synthetic biology methods are expected to facilitate the reliable production of leech-derived molecules like hirudin without relying on natural leech populations, thereby enhancing scalability and reducing regulatory and ethical issues. Increasing focus on this area, backed by recent recombinant production research and clinical examples, would strengthen the argument for leeches as a sustainable source of medicinal compounds [180]. Developments in omics are discovering the molecular composition of leech saliva, which enhances the comprehension of the underlying mechanisms and possible targets. Such development facilitates regulatory acceptance and helps in the rational development of drugs [181]. Recent clinical case reports have documented the successful use of medicinal leeches in relieving venous congestion after reconstructive flap surgery while also noting complications such as bleeding risk and *Aeroman* infection requiring antibiotic prophylaxis and close monitoring [182]. The promise lies in the provision of new drug delivery systems, such as nanoparticles and hydrogels, to increase the targeted delivery and bioavailability of leech bioactives and reduce their side effects [183]. Academia, industry and regulators must work together to develop adaptive science-based frameworks to guarantee patient safety. Also, sustainable harvesting and novel production techniques, such as cell culture and synthetic manufacturing, are important in the standardization of therapies and addressing ethical and environmental concerns [184]. In general, the combination of biotechnology, improved molecular characterization and emerging regulatory science can provide a way to achieve full utilization of the therapeutic potential of leech-derived bioactive compounds in a safe, effective and ethically sound way [33].

## 8. Conclusions

Leech-derived bioactive compounds are a valuable source of molecules with anticoagulant, anti-inflammatory, antimicrobial, and tissue-regenerative properties, showing activity in experimental and some clinical contexts. Their proven use in reconstructive microsurgery highlights their therapeutic importance, while ongoing biochemical and omics research continues to discover new potentially useful molecules. However, evidence for many applications, such as treating cancer, diabetes, and supporting regeneration, is still preliminary and mainly based on in vitro or preclinical studies. Challenges include variability among leech species and extraction techniques, safety issues like bleeding and infection, and the need for standardization and regulatory review. Advances in recombinant production, proteomics, transcriptomics, and targeted delivery are improving consistency and scalability, yet more controlled clinical trials are needed before wider therapeutic use is justified. Collaboration among researchers, clinicians, and regulators is essential to validate effectiveness, ensure safety, and develop sustainable manufacturing. Overall, leech-derived bioactives are a promising but emerging area of biomedical research that requires cautious optimism and thorough investigation.

## Figures and Tables

**Table 1:** Major bioactive compounds from leech salivary secretions, their sources, extraction methods and functional domains.

Compound	Sources	Extraction Method	Molecular Weight	Structural Class	Functional Domain	References
Hirudin	*Hirudo verbana*	Starvation and regurgitation	7 kDa	Peptide	Thrombin-binding	[43]
Eglin C	*Hirudo medicinalis*	Salivary and milking	8 kDa	Protein	Trypsin/chymotrypsin	[38]
Calin	*Hirudo medicinalis*	Manual milking	5 kDa	Protein	Platelet inhibitor	[44]
Bdellin	*Hirudo medicinalis*	Gland extraction	8 kDa	Protein	Serine protease inhibitor	[45]
Destabilase	*Hirudo verbana*	Starvation and regurgitation	20 kDa	Enzyme	Isopeptidase	[46]
Saratin	*Hirudo medicinalis*	Salivary gland extraction	12 kDa	Protein	Platelet aggregation	[47]
Hyaluronidase	*Hirudo verbana*	Regurgitation	40 kDa	Enzyme	ECM degradation	[48]
Factor Xa inhibitor	*Whitmania pigra*	Acidic extraction	11 kDa	Peptide	Coagulation inhibitor	[49]
Leech Antistasin	*Hirudo nipponia*	Regurgitation and lyophilization	15 kDa	Protein	Coagulation cascade	[50]

## Data Availability

Not applicable. No new data were created or analyzed in this study.

## Abbreviations

The following abbreviations are used in this manuscript:

APTT	Activated Partial Thromboplastin Time
TT	Thrombin Time
PT	Prothrombin Time
LC-MS/MS	Liquid Chromatography with Tandem Mass Spectrometry
SEM	Scanning Electron Microscopy
SDS-PAGE	Sodium Dodecyl Sulfate–Polyacrylamide Gel Electrophoresis
BLAST	Basic Local Alignment Search Tool
IC50	Half-maximal Inhibitory Concentration
EC50	Half-maximal Effective Concentration
MIC	Minimum Inhibitory Concentration
MBC	Minimum Bactericidal Concentration
VAS	Visual Analog Scale
CRP	C-Reactive Protein
ESR	Erythrocyte Sedimentation Rate
CRF	Chronic Renal Failure
VSMCs	Vascular Smooth Muscle Cells
VEGF	Vascular Endothelial Growth Factor
HIF-α	Hypoxia-Inducible Factor-alpha
DLL4	Delta-like Ligand 4
TNF-α	Tumor Necrosis Factor-alpha
COX	Cyclooxygenase
iNOS	Inducible Nitric Oxide Synthase
Arg-1	Arginase-1
H&E	Hematoxylin and Eosin
CCK-8	Cell Counting Kit-8
TLR4	Toll-like Receptor 4
LPS	Lipopolysaccharide
LTA	Lipoteichoic Acid
AGE	Advanced Glycation End Products
RAGE	Receptor for Advanced Glycation End Products
SRC	Sarcoma (proto-oncogene tyrosine-protein kinase)
TCA cycle	Tricarboxylic Acid Cycle (Krebs cycle)
LSE	Leech Salivary Extract
LLSE	Lyophilized Leech Saliva Extract
MLT	Medical Leech Therapy
HUVEC	Human Umbilical Vein Endothelial Cell
EC-EX	Endothelial Cell-Derived Exosome
THR	Thrombin
HSTX	Haemadipsa sylvestris Toxin
Hiru	Hirudin
rHmAIF-1	Recombinant Hirudo medicinalis Allograft Inflammatory Factor-1
NaV	Voltage-gated Sodium Channel
JBI	Joanna Briggs Institute
